# Opposing Effects of Zac1 and Curcumin on AP-1-Regulated Expressions of S100A7

**DOI:** 10.1371/journal.pone.0144175

**Published:** 2015-12-03

**Authors:** Yu-Wen Chu, Shu-Ting Liu, Hsiao-Chun Cheng, Shih-Ming Huang, Yung-Lung Chang, Chien-Ping Chiang, Ying-Chun Liu, Wei-Ming Wang

**Affiliations:** 1 Graduate Institute of Medical Sciences, National Defense Medical Center, Taipei, Taiwan, ROC; 2 Department of Pharmacy, Taichung Veterans General Hospital, Taichung, Taiwan, ROC; 3 Biochemistry Department, National Defense Medical Center, Taipei, Taiwan, ROC; 4 School of Nursing, National Defense Medical Center, Taipei, Taiwan, ROC; 5 School of Nursing, National Yang-Ming University, Taipei, Taiwan, ROC; 6 Department of Dermatology, Tri-Service General Hospital, National Defense Medical Center, Taipei, Taiwan, ROC; CNRS-University of Toulouse, FRANCE

## Abstract

ZAC, an encoding gene mapped at chromosome 6q24-q25 within PSORS1, was previously found over-expressed in the lower compartment of the hyperplastic epidermis in psoriatic lesions. Cytokines produced in the inflammatory dermatoses may drive AP-1 transcription factor to induce responsive gene expressions. We demonstrated that mZac1 can enhance AP-1-responsive S100A7 expression of which the encoding gene was located in PSORS4 with HaCaT keratinocytes. However, the mZac1-enhanced AP-1 transcriptional activity was suppressed by curcumin, indicating the anti-inflammatory property of this botanical agent and is exhibited by blocking the AP-1-mediated cross-talk between PSORS1 and PSORS4. Two putative AP-1-binding sites were found and demonstrated to be functionally important in the regulation of S100A7 promoter activity. Moreover, we found curcumin reduced the DNA-binding activity of AP-1 to the recognition element located in the S100A7 promoter. The S100A7 expression was found to be upregulated in the lesioned epidermis of atopic dermatitis and psoriasis, which is where this keratinocyte-derived chemoattractant engaged in the pro-inflammatory feedback loop. Understanding the regulatory mechanism of S100A7 expression will be helpful to develop therapeutic strategies for chronic inflammatory dermatoses via blocking the reciprocal stimuli between the inflammatory cells and keratinocytes.

## Introduction

Human keratinocytes have been widely accepted as an important player in the cutaneous immune system because they provide a physical barrier through a fine-tuned differentiation process and act as an important reservoir for the production of various important antimicrobial peptides (AMPs) [1,2]. On the other hand, the keratinocyte-derived AMPs can also participate in the aforementioned barrier formation or inflammation elicited by environmental insults despite their intrinsic antimicrobial properties. S100A7, also named psoriasin, is a good example [3]. This 11.4 kDa cytoplasmic and secreted polypeptide can protect the skin from the infection caused by *E*. *coli* [4,5]; and it is also an important molecule involved in the construction of an impermeable skin barrier [3,6–8]. S100A7 was first found overexpressed in psoriatic scales [9,10], but further studies have demonstrated that a variety of inflammatory dermatoses and cancers actually exhibited up-regulated an S100A7 expression [7,8,11]. Therefore, it has been postulated that a better understanding of the regulation on the expression of AMPs such as S100A7 may help to provide alternative resolutions for unmet needs in the treatment of inflammatory skin diseases and cancers [12–15].

S100A7 is a potent chemotaxin that has thoroughly engaged in a pro-inflammatory feedback loop which is important in the pathogenic process of human disorders including psoriasis, atopic dermatitis and breast cancer [7,11]. The expression of S100A7 can be up-regulated by cytokines such as IL-17, IL-22, TNF-α, oncostatin-M, IL-6, among others [8,16–18]. Vitamin D analog calcipotriol has been demonstrated useful to disrupt the S100A7-driven pro-inflammatory feedback loop but the underlying molecular mechanism remains elusive [12]. It has been demonstrated that the S100A7 gene is regulated by an activator protein-1 (AP-1)-responsive promoter [19,20]. AP-1 is a crucial transcription factor involved in the expression of many cytokines [21] and in the expression of differentiation-dependent hallmarks of epidermal keratinocytes [22–25]. Interestingly, the transcriptional activity of AP-1 can be regulated by many agents including phorbol ester (PMA) and curcumin [26,27], a botanical derivative that was previously used in some traditional medications and a clinical trial for psoriasis treatment [28–30]. Our previous work has demonstrated that zinc-finger protein, Zac1, which regulates apoptosis and cell cycle arrest 1, physically interacts with AP-1 protein, and enhances the expression of AP-1 regulated genes [31]. Surprisingly, ZAC (the human counterpart of mouse Zac1) has already been shown over-expressed in psoriatic plaques but its functional role remains unknown on the pathogenesis of psoriasis [32].

Overall, S100A7 promoter is responsive to AP-1, of which the transcriptional activity can be fine-tuned by various regulatory extra- and intra- cellular factors [33]. Although evidence supported that the transcriptional activity of AP-1 can be up-regulated by Zac1 but down-regulated by curcumin, the cross-talk between Zac1 and curcumin on the AP-1 regulated S100A7 expression remains largely unknown. Therefore, we began to set up experiments to explore the underlying molecular mechanism of AP-1-regulated S100A7 expressions.

## Materials and Methods

### Cell culture, luciferase reporter assay and chemicals

HaCaT cells were grown in DMEM added with fetal bovine serum pre-treated by 10% charcoal/dextran (Life Technologies, Grand island, NY, USA). Transient transfection (jetPEI, PolyPlus-transfection, Illkirch, France) and luciferase reporter assays (Promega, Madison, WI, USA) were performed in 24-well culture dishes as previously described [34,35]. Total transfected DNA was adjusted to 1 μg by adding the required amounts of the respective empty vector. The luciferase activity detected in the extract prepared from transfected HaCaT cells is expressed in relative light units (RLU) and presented as the mean carrying standard deviation from three independent transfected cultures. We purchased curcumin from the Cayman Chemical Company (Ann Arbor, MI, USA).

### Cell viability assay

Cells were seeded in 96-well culture plates and allowed to grow for 24 h. HaCaT cells were then treated by the experimental chemicals in fresh culture medium for the indicated durations. MTT (3-(4,5-dimethylthiazol-2-yl)-2,5-diphenyltetrazolium bromide) solution (0.5 mg/ml in PBS) was added to each well of the aforementioned culture plates, which were further incubated for 2 h at 37°C. Dimethylsulfoxide (DMSO) (150 μl), the solubilizing agent, was then added and subjected to measure respective absorbance at 540 nm using an ELISA plate reader (Multiskan EX, Thermo Fisher Scientific, Waltham, MA, USA). HaCaT cells treated with culture media containing no compounds were used at a control and set as 100% cell survival.

### Measurement of Reactive Oxygen Species (ROS) Assay

Intracellular ROS levels were determined using the fluorescent marker 2’,7’-dichlorofluorescin diacetate (DCFH-DA; D6883; Sigma-Aldrich, MO, USA), according to the manufacturer’s instructions. Briefly, the cells were seeded in 6-well culture plates and treated with indicated drugs for 16 h. After incubated, we stained live cells with 10 μM DCFH-DA for 30–60 min at 37°C, harvested the cells, and washed twice with PBS. The cells were then subjected to FACS, and the fluorescence intensity analysis was performed using a FACSCalibur flow cytometer and the Cell Quest Pro software (BD Biosciences, CA, USA).

### Immunoblots

Using RIPA buffer (100 mM Tris-HCl pH 8.0, 150 mM NaCl, 0.1% SDS, and 1% Triton 100) we lysed and prepared cell extracts at 4°C, which were separated by SDS-PAGE and then immunoblotted by using antibodies against S100A7 (Imgenex, San Diego, CA, USA), HA antibody (3F10, Roche, Basel, Switzerland); and c-Fos and ACTN (Santa Cruz Biotechnology, Santa Cruz, CA, USA) for further interpretation.

### Plasmids

Reporter gene S100A7(-743/+1)-LUC has been previously described [19]. The S100A7(-743/+1)-LUC vectors carrying various AP-1 binding site-directed mutagenesis (AP-1 1M and/or 2M) were made using the Promega Gene Edit Kit. The mutant sequence information of M1 site was 5’-tacgtaa-3’ and M2 site was 5’-tacgtaa-3’ [36]. Distinct HA-tagged AP-1 proteins encoded by the pSG5.HA vector have been previously described [31].

### Reverse transcriptase-polymerase chain reaction (RT-PCR)

The growing HaCaT cells were used for total RNA extraction by TRIsure reagent (BIOLINE, London, UK) following the manufacturer’s instructions. Total RNA (1μg) was subjected to reverse transcription for 60 min at 37°C using MMLV reverse transcriptase (Epicentre Biotechnologies, Madison, WI, USA). In the linear range (30 cycles), PCR was performed with primers specific for *S100A7* and *GAPDH*. The DNA sequences of PCR primers used for the amplification of anticipated genes, S100A7 and GAPDH have been previously described [19,20]. The thermocycling conditions for PCR were as following: single run for 5 m at 95°C, followed by 30 cycles of sequential steps including 45 s at 95°C, 30 s at 55°C, and 40 s at 72°C. Amplified DNA products were subjected to the separation by 1.2% agarose gel electrophoresis and staining with ethidium bromide for visualization.

### Quantitative PCR

Total RNAs were isolated using the TRIsure reagent according to the manufacturer’s instructions. One microgram of the total RNA samples was subjected to reverse transcription (RT) according to cDNA protocols (Epicentre Biotechnologies, MI, USA). The cDNA products were used immediately for SYBR green (Applied Biosystems, CA, USA) real-time RT-PCR. Real-time RT-PCR was done using ABI Prism 7500 Sequence Detection System (Applied Biosystems) with Fast SYBR Green Master Mix (Applied Biosystems). PCR amplification consisted of an initial denaturation step (95°C for 3 m) and 40 cycles of denaturation (95°C for 5 s), annealing and extension (60°C for 35 s). The S100A7 primers used were as follows: forward 5’-ACGTGATGACAAGATTGAGAAGC-3’ and reverse 5’-GCGAGGTAATTTGTGCCCTTT-3’. The GAPDH primers were forward: 5’- CCTCCCGCTTCGCTCTCTG-3’ and reverse: 5’-GCGCCCAATACGACCAAATC-3’ as an internal control.

### Modified DAPA (DNA-affinity precipitation assay)

Two biotin-labeled double-stranded DNA fragments in which the oligonucleotide sequence of the sense strand primer as 5’-ggattTGATTCAggcttttc-3’ containing wild-type AP1-1 site and 5’-ggattTACGTAAggcttttc-3’ containing mutated AP1-1 site were used in the assays. The nuclear extracts were prepared as previously described [37]. DAPA binding buffers containing 10 mM Tris/HCl, pH 7.5, 50 mM KCl, and 1 mM DTT (dithiothreitol) was used for incubation with HaCaT nuclear extracts, which were pre-incubated with the nonspecific Poly(dI-dC) (dI-dC) competitor and followed by incubation with the aforementioned double-stranded oligonucleotides. After the incubation, anti-c-Fos antibody was added for incubation to the reaction mixture. The anticipated protein–DNA–agarose complex was then processed as previously described [38]. Detection was performed adding streptavidin–horseradish peroxidase (Panomics, Santa Clara, CA, USA) and Western blot analysis.

### ChIP (Chromatin immunoprecipitation) analysis

The ChIP assay was performed using Pierce^TM^ Magnetic ChIP Kit (ThermoFisher Scientific, MA, USA), according to the manufacturer’s instructions. In brief, HaCaT cells were treated with 10 μM curcumin or ethanol (vehicle) for 24 hrs, and then crosslinked by 1% formaldehyde for 10 min with following neutralization by adding glycine to a final concentration of 0.1 M for 5 min at room temperature. After washing twice with cold PBS, cells were harvested in ice-cold PBS (containing protease inhibitor cocktail), and then the cell pellets were suspended in cold membrane extraction lysis buffer containing protease/phosphatase inhibitors. After 10 min incubation on ice, nuclei were harvested in MNase Digestion Buffer and re-suspended in IP Dilution Buffer containing protease/phosphatase inhibitors, and then sonicated to achieve fragmentation of DNA to 200–1000 base pairs in length. Immunoprecipitations were carried out using anti-c-Jun (sc-1694; Santa Cruz Biotechnology) and anti-c-Fos (sc-253; Santa Cruz Biotechnology). Normal rabbit IgG, included in Pierce^TM^ Magnetic ChIP Kit, was used in the negative control IP experiments. The DNA sequence of S100A7 PCR primers (330 bp) was as following: forward strain 5’-CTTCTGTGAGGGGCTGACCA-3’ and reverse strain 5’- TCTATGACCCCCACCGCTGA-3’. The negative control (control) primers (325 bp) were forward: 5’-CAGAGGGTGAGGGTGATCTG-3’ and reverse: 5’-TACTCTGTCCTCAGCCCTCC-3’.

### Statistical methods

The statistical analyses were performed using an independent Student's t-test. All *p* values less than 0.05 were considered statistically significant.

## Results

### Expression of S100A7 in HaCaT cells was inhibited by curcumin but enhanced by Zac1

**Fig 1:**
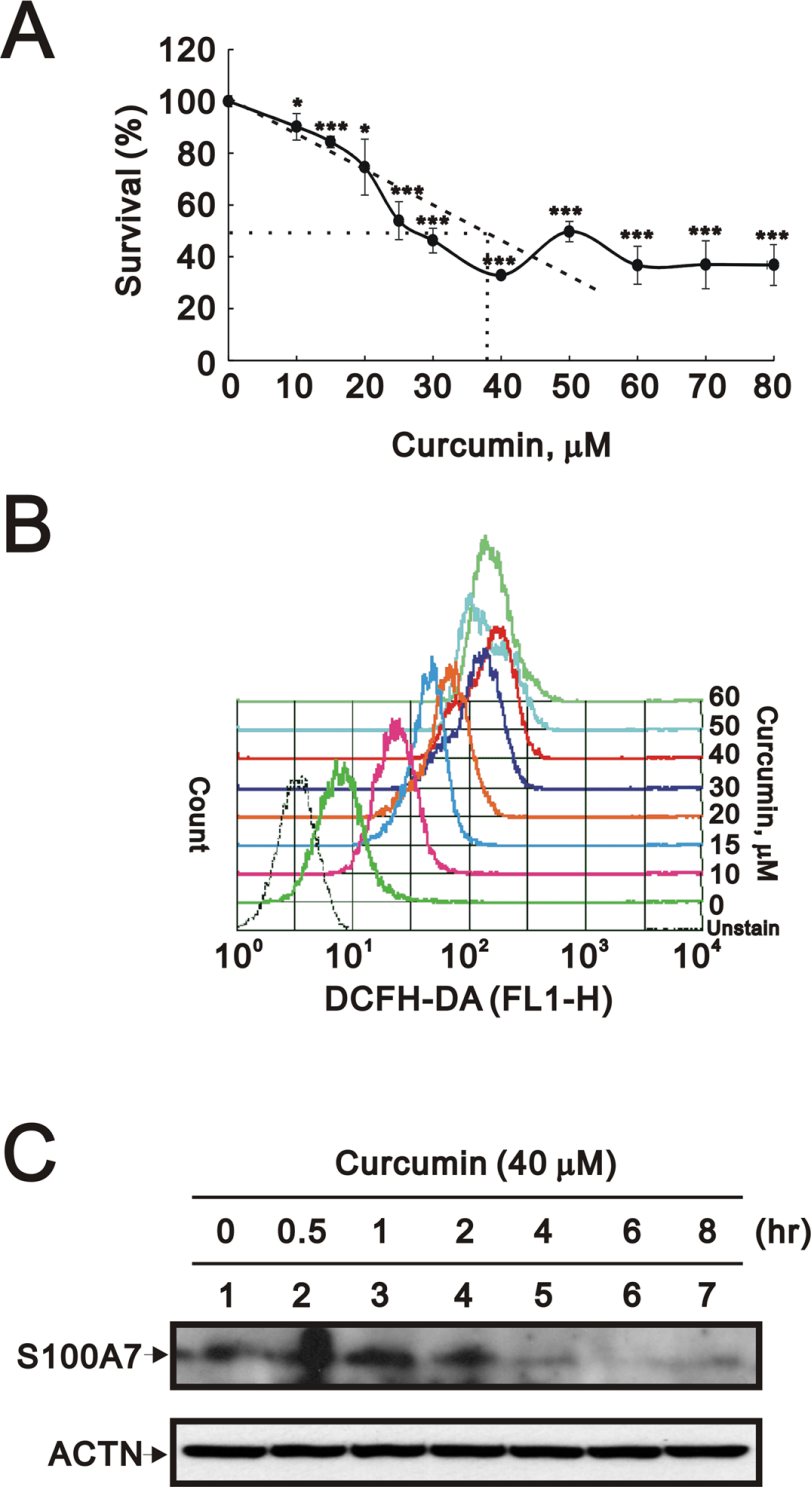
S100A7 expression is inhibited by curcumin in a time-dependent manner. (**A**) IC50 of curcumin was detected using MTT assay for HaCaT cell survival rate after 24 h of incubation with increasing dosage of curcumin. (B) ROS analysis was measured after 16 h of incubation with increasing dosage of curcumin. (**C**) Western blotting using specific antibody against S100A7 revealed that the S100A7 expression in HaCaT keratinocytes was inhibited in a time-dependent manner after treatment by curcumin. Results (A, B and C) are representative of two independent experiments. All *p* values less than 0.05 were considered statistically significant. * *p*<0.05; **p<0.01; ***p<0.005.

**Fig 2:**
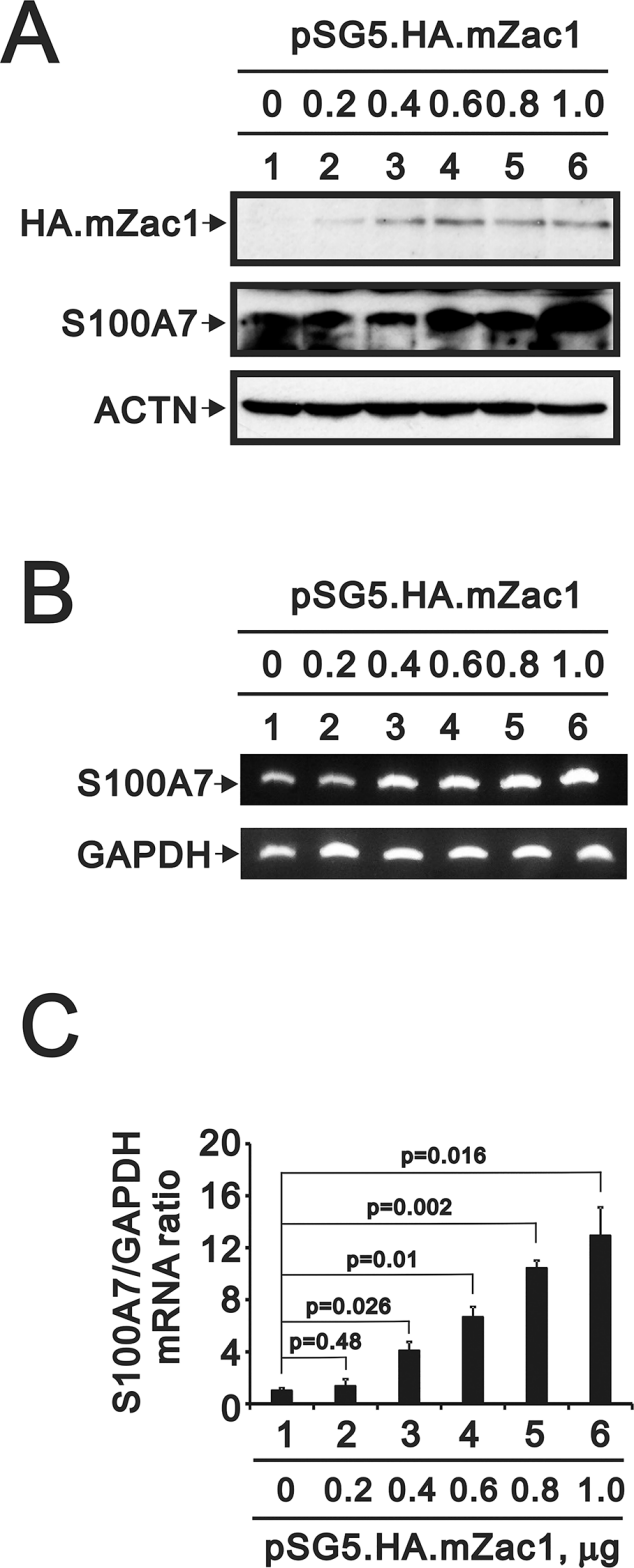
S100A7 expression is increased by Zac1. (**A**) HaCaT cells were transiently transfected with increasing dose of plasmid for over-expression of exogenous HA-tagged mZac1. Immunoblotting using specific antibodies against HA-tag or S100A7 was performed to detect the protein level of over-expressed HA.mZac1 and endogenous S100A7 in lysates from cultured HaCaT keratinocytes. The activated expression of S100A7 mRNA transcript was analyzed by general RT-PCR (**B**) and quantitative real-time RT- PCR (**C**). Results (A and B) are representative of two independent experiments. These data (C) are the average of three experiments (mean ± SD; n = 3). All *p* values less than 0.05 were considered statistically significant.

Using the MTT assay, we first detected the IC50 of curcumin by treating HaCaT cell culture for 24 h and the result was around 38 μM (Fig 1A). In addition, we added the dose-dependent effect of curcumin on the reactive oxygen species (ROS) production in HaCaT cells (Fig 1B). We observed the similar reduced pattern at the higher curcumin dosage (around 40–50 μM) in the MTT and ROS analysis. Then, we checked the expression of S100A7 in HaCaT cell culture in the absence or presence of 40 μM curcumin. We found the protein level of S100A7 was reduced in a time-dependent manner (Fig 1C). Interestingly, when we over-expressed Zac1 in HaCaT cells, the amount of S100A7 was increased accordingly at either the protein (Fig 2A) or the mRNA transcript level (Fig 2B). We further quantitatively and statistically analyzed the effect of over-expressed Zac1 on the S100A7 gene expression (Fig 2C).

### S100A7 promoter activity activated by AP-1 can be further enhanced by Zac1

**Fig 3:**
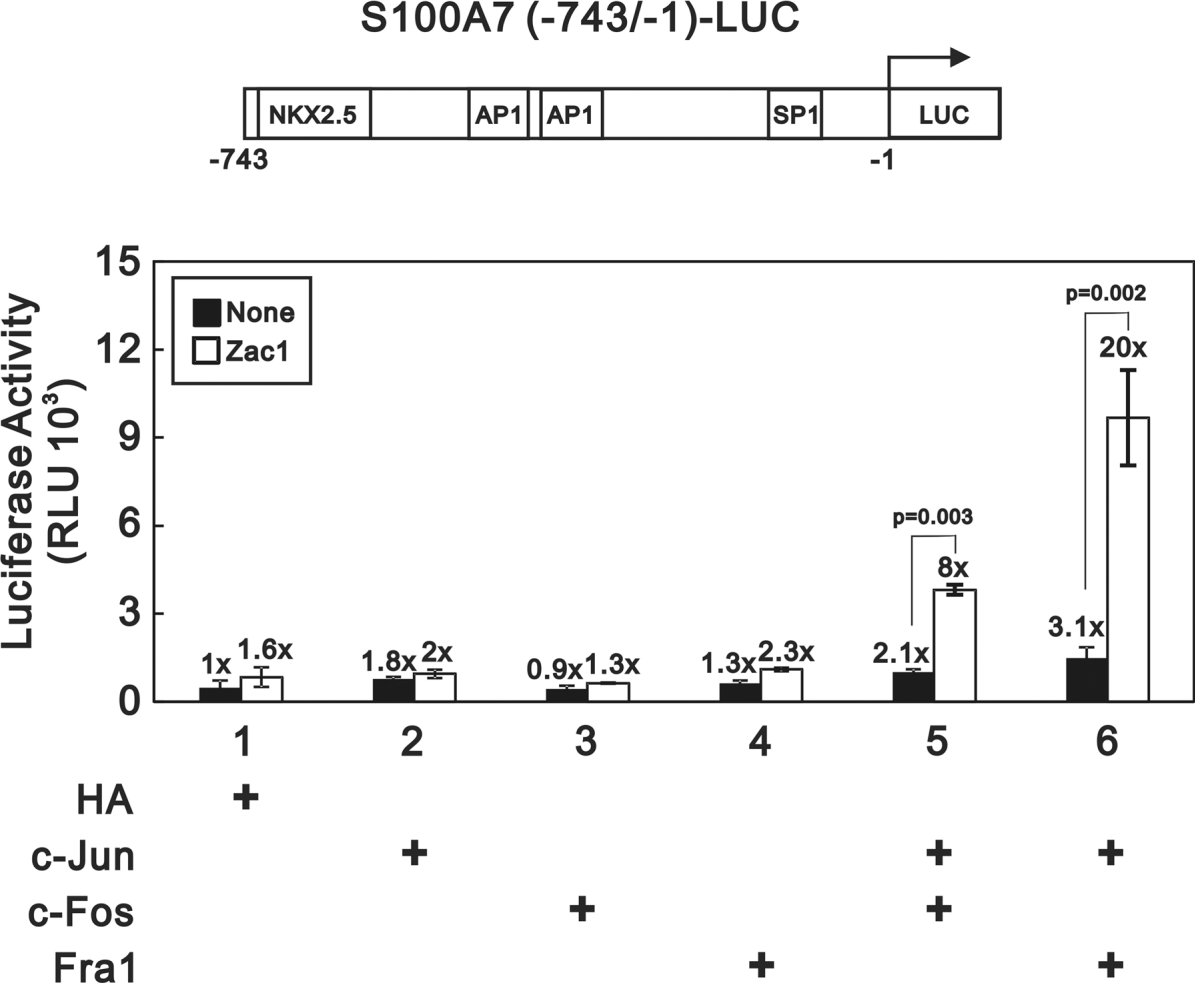
Zac1 significantly enhances the S100A7 promoter activity driven by heterodimeric AP-1 complexes. The schematic presentation of the luciferase reporter driven by S100A7 promoter containing two putative AP-1 binding sites is shown at the upper panel. Although previously we have demonstrated that Zac1 can physically interact with each of c-Jun, c-Fos and Fra-1, the S100A7 promoter activity can only be mildly enhanced by over-expressed Zac1 itself (lane 1; compare open to closed bar) or together with co-expressed c-Jun, c-Fos, or Fra-1 (compare lanes 2–4 to lane 1; open bars to closed bars), respectively. However, the S100A7 promoter activity driven by heterodimeric AP-1 complexes formed by c-Jun/c-Fos or c-Jun-Fra-1 paring is significantly enhanced by co-expressed Zac1 (lanes 5 and 6; compare open to closed bars). These data are the average of three experiments (mean ± SD; n = 3). All *p* values less than 0.05 were considered statistically significant.

Previously, we reported that Zac1 can physically interact with the c-Jun, c-Fos and Fra-1 subunit of AP-1 protein, whose transcriptional activity would also be enhanced by Zac1 in HeLa cells [31]. We have also demonstrated that c-Jun/c-Fos heterodimeric AP-1 can activate the S100A7 promoter activity in human keratinocytes [19]. Then, we started to examine whether the AP-1 activated S100A7 promoter activity would be enhanced by Zac1 in HaCaT cells. Using a luciferase assay driven by S100A7 promoter, we first demonstrated the promoter activity can be activated by both c-Jun/c-Fos and c-Jun/Fra-1 heterodimeric AP-1 at 2.1- and 3.1- fold, respectively (Fig 3; compare lanes 5–6 to lane 1, closed bar). The aforementioned AP-1 activated promoter activity was further enhanced by exogenous Zac1 over-expressed in HaCaT cells (Fig 3; compare lanes 5–6 to lane 1, open bar).

### The AP-1 transcriptional activity stimulated by Zac1 was inhibited by curcumin

**Fig 4:**
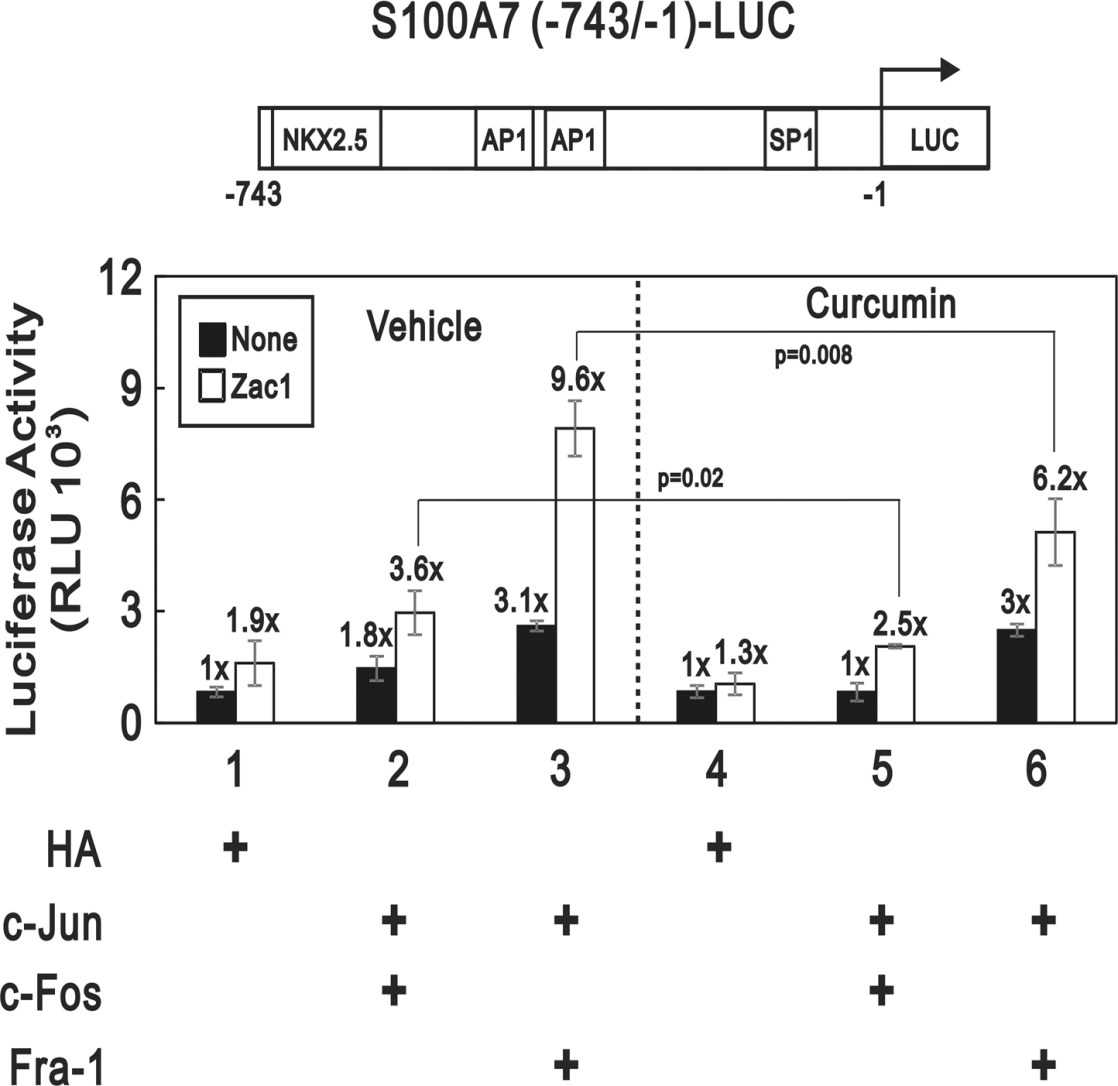
The AP-1 transcriptional activity stimulated by Zac1 was inhibited by curcumin. The schematic presentation of the luciferase reporter driven by S100A7 promoter containing two putative AP-1 binding sites is shown at the upper panel. Although curcumin is a well-known AP-1 inhibitor, the S100A7 promoter driven by co-expressed c-Jun/c-Fos but not c-Jun/Fra-1 heterodimer is abolished by curcumin treatment (compare lanes 5 to 2 or lanes 6 to 3, closed bars). Instead, the Zac1-enhanced S100A7 promoter activity driven by either c-Jun/c-Fos or c-Jun/Fra-1 heterodimeric AP-1 complexes is diminished by curcumin c-Jun/c-Fos but not c-Jun/Fra-1 (compare lanes 5 to 2 or lanes 6 to 3, open bars). Interestingly, the S100A7 promoter activated by over-expressed Zac1 alone is also inhibited by curcumin (compare lane 4 to 1, open bars). These data are the average of three experiments (mean ± SD; n = 3). All *p* values less than 0.05 were considered statistically significant.

We further asked whether the S100A7 promoter activity activated by AP-1 in the absence or presence of exogenous Zac1 would be diminished by curcumin because the expression of S100A7 in keratinocytes was shown to be activated by Zac1 (Fig 2) but inhibited by curcumin (Fig 1). Fig 4 shows that curcumin selectively inhibits the transcriptional activity of c-Jun/c-Fos but not c-Jun/Fra-1 in the absence of exogenous Zac1 (Fig 4; closed bars). However, the stimulatory effect exhibited by Zac1 on either c-Jun/c-Fos or c-Jun/Fra-1 heterodimeric AP-1 complex was significantly inhibited by curcumin (Fig 4; open bars).

### Two putative AP-1 binding sites were functionally important for S100A7 promoter activity driven by c-Jun/c-Fos and c-Jun/Fra-1 heterodimers

**Fig 5:**
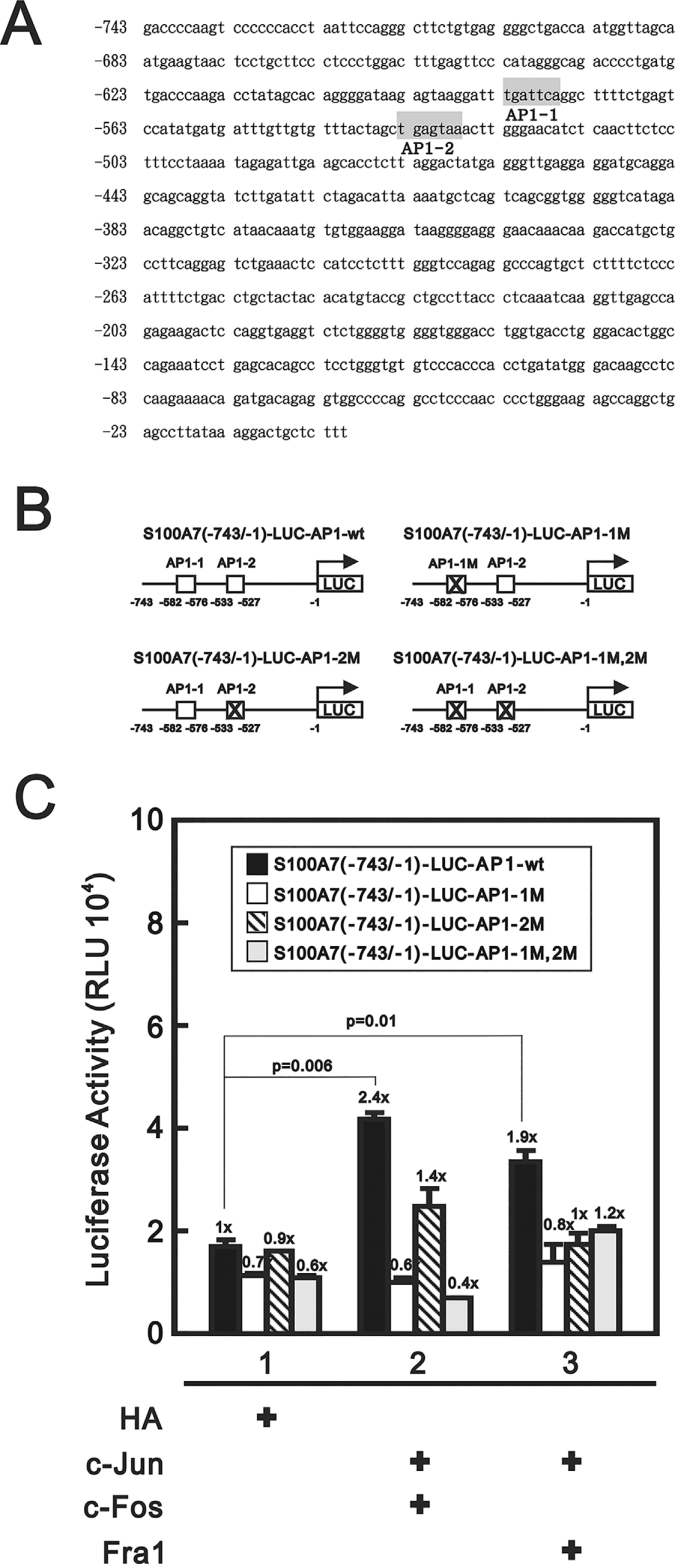
Differential functional roles for two AP1 binding sites of S100A7. (**A**) The DNA sequence of S100A7 promoter (GenBank accession number: AF050167) is shown and two putative AP-1 binding sites are framed by gray shadow. (**B**) The schematic presentation of the luciferase reporters driven by S100A7 promoter containing different mutated AP-1 binding sites is indicated. (**C**) The luciferase reporter assays were performed using aforementioned individual construct in the absence or presence of either c-Jun/c-Fos or c-Jun/Fra-1 heterodimeric AP-1 complexes. Both the basal activity (lane 1, open bar) and AP-1-activated promoter activities (lanes 2 and 3, open bars) are inhibited by AP1-1 site mutation, indicating the essential role of this AP-1 binding site. On the other hand, the S100A7 promoter containing mutated AP1-2 site can still be activated by heterodimeric c-Jun/c-Fos instead of c-Jun/Fra-1 (compare lanes 2–3 to lane 1, dashed bars). The result indicated that the functional role of AP1-2 site is less than AP1-1 site for c-Jun/c-Fos; however, the two putative AP-1 binding are both important for c-Jun/Fra-1 to activate S100A7 promoter. Surprisingly, c-Jun/Fra-1 can activate the S100A7 promoter containing two mutated AP-1 binding sites (compare lanes 2–3 to lane 1, light grey bars), indicating that the S100A7 promoter may be activated by heterodimeric c-Jun/Fra-1 AP-1 complex with some unexplored mechanism. These data (C) are the average of three experiments (mean ± SD; n = 3). All *p* values less than 0.05 were considered statistically significant.

We continuously investigated the functional role of AP-1 binding sites because S100A7 promoter activity can be driven by AP-1 transcription factors. First, we looked for putative AP-1 binding sites by analyzing the DNA sequence of S100A7 promoter (GenBank accession number: AF050167) using the TFSEARCH website (http://www.cbrc.jp/research/db/TFSEARCH.html). Two putative AP-1 binding sites numbered as AP1-1 and AP-2 were located at -582/-576 and -533/-527 upstream of the transcription start site (Fig 5A). The AP1-1 site has been demonstrated to be functional in activation of the S100A7 promoter [20]. The AP1-2 site, TGAGTAA, is a non-canonical AP-1 site and has been found functionally important for the activation of HPV-11 E6 promoter activity [36]. Moreover, this AP-1 binding element has been clearly demonstrated to be physically bound by various recombinant human AP-1 complexes [22,39]. We used site-directed mutagenesis (Fig 5B) to analyze the functional role of each site for activation of S100A7 promoter activity. For an AP-1 complex composed of c-Jun/c-Fos subunits, the mutagenesis performed on the AP1-1 site almost abolished the promoter activity driven by this heterodimer. However, residual activity was revealed when AP1-2 site was mutated alone but abolished again when AP1-1 site was mutated in combination (Fig 5C). The aforementioned results indicate that AP1-1 site is more important than AP1-2 site for c-Jun/c-Fos heterodimers to drive S100A7 expression. On the other hand, individual mutagenesis on either AP1-1 site or AP1-2 site abolished the S100A7 promoter activity driven by c-Jun/Fra-1, which indicates both sites are equally important for c-Jun/Fra-1 heterodimer to drive the S100A7 expression.

### Curcumin prevented the binding of AP-1 from its functional site within S100A7 promoter

**Fig 6:**
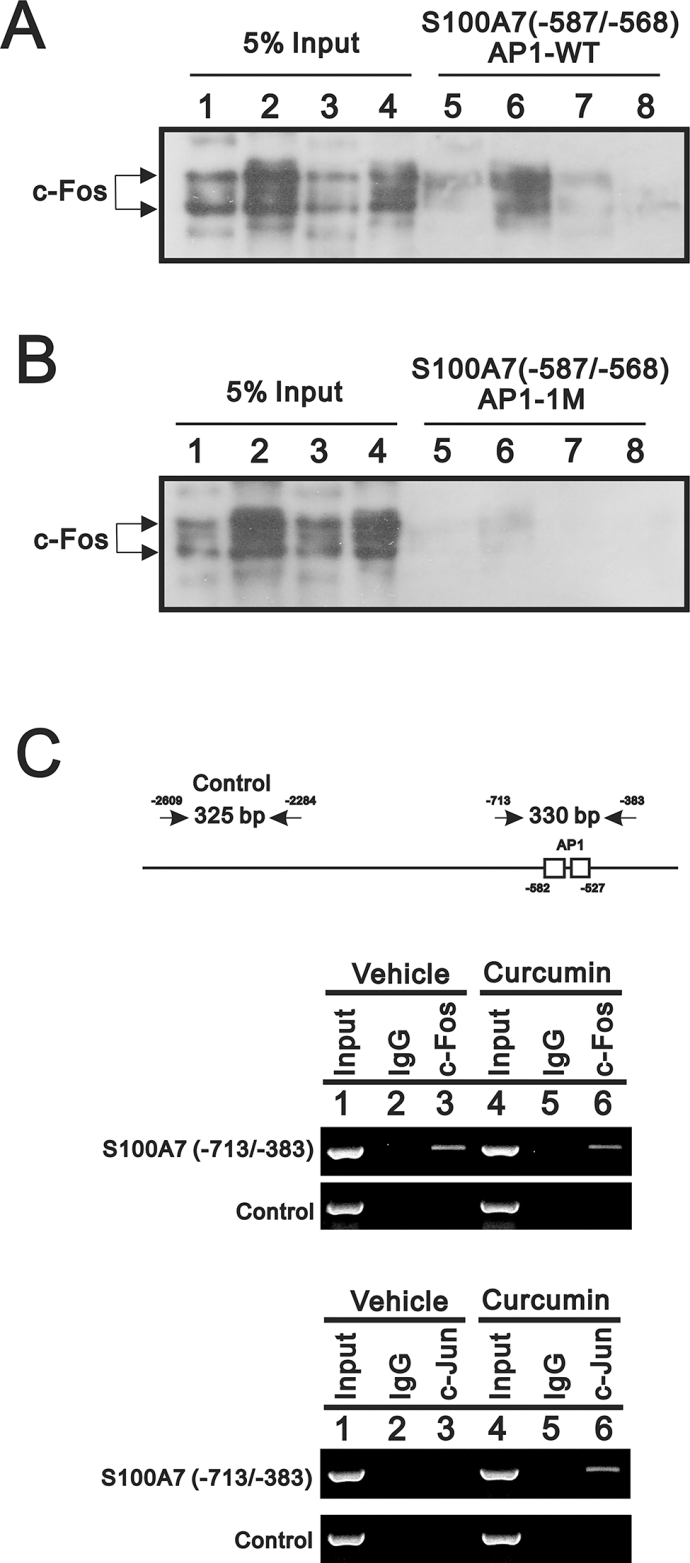
Curcumin prevented the binding of AP-1 to the functionally important AP1-1 site carried in the S100A7 promoter region. Biotin-labelled DNA fragments compatible with the sequence as -587 to -568 nucleotides of S100A7 promoter carrying either wild-type or mutated AP1-1 binding site were used to attach AP-1 complexes contained in the nuclear extracts prepared from HaCaT cells pre-incubated with PMA (lane(s) 2 and 6), curcumin (lane(s) 3 and 7) or combinations (lane(s) 4 and 8). The DNA-AP-1 complexes were immunoprecipitated by anti-c-Fos antibody than further processed for western blotting analysis since AP1-1 site acts as the predominant functional site for c-Jun/c-Fos to activate S100A7 promoter. (**A**) The presence of c-Fos at the wild-type AP1-1site was analyzed by western-blotting using anti-c-Fos antibody. Shown in lane(s) 1–4 are the 5% input of elute derived for each indicated condition. The amount of c-Fos recruited to the AP1-1 site is increased by the AP-1 stimulant PMA (comparing lane 6 to lane 1) but reduced by curcumin (compare lane 7 to lane 5). Moreover, the stimulatory effect derived from PMA treatment is also inhibited by curcumin (compare lane 8 to lane 6). (**B**) The presence of c-Fos at the mutated AP1-1site was also analyzed. Obviously, the recruitment of AP-1 is abolished by the mutagenesis performed on AP1-1 site (compare lane(s) 5–8 to lane 1~4). (**C**) The two PCR products from respective primer pairs covered the DNA fragment containing two AP-1 sites and indicated control region of endogenous S100A7 promoter were analyzed in the ChIP analysis. The different effect of curcumin were found using the respective antibody against different subunit of AP-1 complex, such as phosphorylated c-Fos and c-Jun. The results indicated more c-Jun-containing complexes will be recruited by curcumin for AP-1 sites occupancy in the endogenous S100A7 promoter. Results (A, B and C) are representative of two independent experiments.

We want to know whether curcumin can prevent AP-1 from binding to the functionally important DNA-binding element. We started to check on the binding affinity in the absence or presence of curcumin. We decided to analyze the binding affinity by checking the binding amount of c-Fos on probes carrying either wild-type or mutated AP1-1 site because we found that the AP1-1 site is important for both heterodimeric c-Jun/c-Fos and c-Jun/Fra-1; in particular, it functions as a major site for c-Jun/c-Fos to drive S100A7 promoter activity. PMA was used as a positive control because it is a well-known AP-1 stimulator able to enhance the DNA-binding activity of AP-1 via PKC signaling pathway [21]. Fig 6A shows that c-Fos is bound by a probe carrying wild-type AP1-1 site and was enhanced by the PMA treatment (compare lane 6 to lane 1). Importantly, the PMA-enhanced binding was almost completely abolished by either curcumin treatment (Fig 6A; compare lane 8 to lane 6) or mutation of AP1-1 site (compare Fig 6B to 6A, lanes 5–8). We further performed ChIP assays to detect the differential occupancy of AP-1 binding sites in the endogenous S100A7 promoter caused by curcumin treatment to HaCaT cells. Interestingly, the accessibility of chromatin fragment containing both AP1-1 and AP1-2 sites by phosphorylated c-Fos was slightly altered by curcumin treatment (Fig 6C). However, curcumin dramatically increased the binding of c-Jun to our predicted AP-1 binding sites of S100A7 promoter, indicating more c-Jun-containing complexes were allowed to get access to this region to compete with c-Fos-containing species for AP-1 binding sites occupancy and possibly caused consequent decreased activity of S100A7 promoter.

## Discussion

The advances in understanding the pathogenic mechanism of multifactorial inflammatory dermatoses such as psoriasis and atopic dermatitis have resulted in significant progress in disease management including the application of various selective biologics for psoriatic patients [40–42]. However, there are still challenges and unmet needs remain [41,43]. In addition to using biologics to prevent susceptible cells from the attack by extracellular cytokines, strategies are developing to target key molecules downstream from the cytokine signaling pathways [44,45]. Moreover, various environmental stimuli including inflammatory cytokines convey messages on transcription factors such as NF-kB and AP-1 thereby triggering corresponding physiological or pathological events. Our study shows that the S100A7 expression can be modulated by targeting AP-1 transcriptional activity, which is promising to perturb the amplified pro-inflammatory feedback loop exhibited in chronic inflammatory dermatoses.

Amplified pro-inflammatory feedback loops have been noticed in psoriasis and atopic dermatitis [40,46,47]. A major and shared question remains debated in clinically distinct diseases and whether they are primary abnormalities resulting from the defective genetic background inherited in the epidermal differentiation complex or from the dysregulated immune reactions [17,48,49]. Moreover, increased S100A7 expression is a common feature shown in skin lesions of both multifactorial diseases [10,50]. The IL-17/IL-22 and other cytokines upregulate the S100A7 expression in keratinocytes, reciprocally the keratinocyte-secreted S100A7 may stimulate the pro-inflammatory cytokines production from infiltrated inflammatory cells or keratinocytes themselves [12,17,51,52]. Interestingly, the S100A7 gene was mapped to chromosome 1q21.2-q22; and the human ZAC gene was shown to reside at chromosome 6q24-q25. Both proteins have been demonstrated overexpressed in psoriatic lesions and our experimental results seem to reflect an interaction between the psoriasis susceptibility loci, PSORS4 and PSORS1 [53]. The gene-gene cross-talk can be mediated by AP-1, and this conversation can be blocked by curcumin via abolishing mZac1-enhanced transcriptional activity of AP-1 and prevent the access of AP-1 transcription factor to the DNA-binding sites located in the promoter of the S100A7 gene.

The regulation of AP-1 transcriptional activity can be fine-tuned at various aspects, including changing the constituted dimeric subunits by different members of the Jun family (c-Jun, JunB, and JunD) and the Fos family (c-Fos, FosB, Fra-1, and Fra-2), interacting with different ancillary proteins, and accessible binding sites composed of canonical or non-canonical DNA-binding sequences that display differential binding affinities, among others [22,33,39,54]. We demonstrated that the inhibitory effect may be differential depending on the dimeric composition of AP-1 subunits although curcumin has been known as an AP-1 inhibitor. Previously, we demonstrated that mZac1 can physically interact with AP-1 and functionally enhanced its transcriptional activity in HeLa cells [31]. We further demonstrated that mZac1 can enhance S100A7 expression activated by AP-1 using human HaCaT keratinocytes, which implies a possible pathogenic role of the over-expressed human ZAC ortholog found in psoriatic skin lesions [32]. Moreover, curcumin abolishes the mZac1 enhanced AP-1 transcriptional activity and possibly exhibits its transcriptional inhibition of S100A7 expression by decreasing the access of AP-1 to the functionally important binding sites found in the promoter region of S100A7 gene as demonstrated in our study.

IL-23/Th17 axis and IL-22 have been shown critical in the chronic progression of psoriasis and atopic dermatitis [17,55]. Interestingly, the expression of IL-23 p19 subunit is also regulated by AP-1 [56]. Considering the amplified pro-inflammatory feedback loop found in intractable inflammatory dermatoses [46,47], we believe the in-depth understanding of regulatory mechanism of S100A7 expression shown in our studies will shed light on the development of AP-1 inhibitors that might contribute to new target therapies for disease management.
